# Feasibility of a videoconferencing-based parent-mediated intervention: a mixed-method pilot study

**DOI:** 10.3389/fpsyg.2024.1450455

**Published:** 2025-01-07

**Authors:** Marie-Maude Geoffray, Maeva Bourgeois-Mollier, Maud Maleysson-Baste, Natacha Gallifet, Sara Dochez, Gaelle Bonis, Agathe Jay, Lucie Jurek

**Affiliations:** ^1^Child and Adolescent Psychiatry Department, Le Vinatier Hospital, Bron, France; ^2^RESHAPE U1290, University of Lyon, Lyon, France; ^3^Baby Lab, ISC, CNRS UMR 5229, Bron, France

**Keywords:** autism spectrum disorder, parent-mediated intervention, PACT, videofeedback, mixed-methods

## Abstract

**Background:**

Autism Spectrum Disorder (ASD) presents early communication and social challenges, necessitating timely and accessible intervention. Pre-school Autism Communication Therapy (PACT), a parent-mediated intervention, empowers parents to facilitate their child’s development. However, accessibility issues often hinder families from accessing evidence-based intervention. This pilot study assessed the feasibility of videoconferencing-based PACT as a precursor for a multicenter randomized controlled trial.

**Methodology:**

A mixed-methods approach integrated quantitative retrospective measures and semi-structured interviews. Participants included children diagnosed with ASD who received PACT, and PACT-trained professionals with videoconferencing experience. Feasibility was assessed through audio and video quality, internet stability, and session length. Professionals’ experiences were analyzed using a qualitative thematic analysis. Autism severity, parent–child interaction, and therapeutic changes were also described.

**Results:**

Nine parent–child dyads and eight PACT therapists were included in the study. Videoconferencing-based PACT intervention proved feasible, with 95.1% of the 41 sessions rated as feasible on the scale. Technical challenges such as audio quality (7.3%) and screen sharing (19.1%) have emerged, which therapists circumvented to maintain intervention quality. Autism severity and parent–child interaction showed positive trends, supported by qualitative findings reporting increased parental confidence and enhanced synchrony. The core components of PACT were successfully adapted to the remote setting.

**Conclusion:**

This pilot study suggests that delivering PACT via videoconferencing is a feasible approach to enhance the accessibility of evidence-based interventions for ASD. Larger-scale research with rigorous controls is required to validate these promising findings. An ongoing multicenter randomized trial aims to address this gap.

## 1 Introduction

Early intervention is essential in Autism Spectrum Disorder (ASD) (Lord et al., 2022). Recent systematic reviews have underlined the efficacy of developmental methods (Trembath et al., 2023). Because parents are closely connected to their children and play a pivotal role in their development, certain developmental approaches, such as Pre-school Autism Communication Therapy (PACT), focus on guiding parents in learning strategies and implementing the approach in daily life (Green et al., 2010; Nevill et al., 2018; Pickles et al., 2016).

Unfortunately, families often lack access to evidence-based interventions, with one to three-year delay in starting early intervention services after diagnosis (Dimian et al., 2021, 2021; Yingling et al., 2018). In France, even within academic centers, the mean age at diagnosis is estimated to be 4.9 years, further delaying access to interventions (Rattaz et al., 2022). Several barriers, described in international literature, contribute to this issue, including long waitlists, distant locations from specialized centers, lack of transportation, and parental time constraints (Balabanovska et al., 2024; Dimian et al., 2021; Jurek et al., 2022; Kanne and Bishop, 2021). These challenges have hindered the implementation of parent-mediated interventions.

Despite being a promising method for improving access to PACT, videoconferencing’s feasibility needs careful evaluation to maintain intervention quality. So far, only a limited number of studies have explored the role of telehealth in delivering videofeedback-based intervention for ASD (Aiello et al., 2022; Alatar et al., 2023). Considering high attrition rates in intervention programs (Chacko et al., 2016), barriers and facilitators of implementation should also be evaluated.

The main objective of the present pilot study was to evaluate the feasibility of videoconferencing PACT in the perspective of undergoing a multicenter randomized controlled study (Jurek et al., 2021). The secondary objective was to describe the evolution of children’s autistic signs and parent–child interactions throughout the intervention.

## 2 Method

The study employed a mixed-methods approach, combining quantitative measures and semi-structured interviews to assess the feasibility and identify barriers and facilitators of the remote intervention. While the primary aim was not to evaluate the intervention’s effectiveness, we also examined its impact on child development and parent–child interactions, observing the progress of the dyad within the new context of video conferencing.

### 2.1 Participants

#### 2.1.1 Therapist population

Professionals trained in PACT were included if they had at least one experience of PACT using videoconferencing (at least six sessions using videoconferencing). Eight therapists were included. They were all women. Three were working as speech therapists, two as nurses, one as a psychologist, one as a psychomotor therapist, and one as a child and adolescent psychiatrist. All therapists had obtained certification in the PACT model, ensuring a fidelity of at least 80% to the model. They were undergoing weekly supervision. Six of them had been using PACT for two years, and two had 4-years of experience. An average of 4.25 families per therapist (range 1–9) had undergone or were currently engaged in PACT at the time of the interview.

#### 2.1.2 Parent–child dyads population

Parent–child dyads were eligible if the children received a diagnosis of ASD in our specialized unit at Le Vinatier, Bron (France), according to DSM-5 (American Psychiatric Association, 2013) and received PACT intervention until January 2021. They were included if they received at least 12 sessions of PACT, with at least half of the sessions being conducted remotely. Eighteen children were eligible. Five were excluded because they did not receive any remote sessions, while four were excluded because they had just started the intervention and had completed fewer than 12 sessions. Nine children (one girl and eight boys) were included in the study. The mean age was 37.7 (+/− 8) months at the beginning of the therapy. The time between ASD diagnosis and PACT was between zero and 16 months. Of the nine children, seven were engaged in speech therapy and one in motor therapy. At baseline, six children had severe or moderately severe ASD symptoms, two had moderate ASD symptoms and one had minimal ASD symptoms, according to Clinical Global Impression–Severity (OSU-CGI-S).

### 2.2 Intervention

PACT is a parent-mediated therapy in which professionals guide caregivers to identify behaviors that promote communication. The intervention and its adaptation to videoconferencing in described in Table 1. The videoconferencing software used was LifeSize, the platform implemented in our institution.

**Table 1:** Pre-school autism communication therapy (PACT) (Aldred et al., 2004; Green et al., 2010).

General aspect of PACT	
Focuses on natural dyadic interactions between parents and children Video-feedback where parents review and discuss recorded parent–child interactions is used Therapist observes, suggests ways to expand shared attention, and guides parents in fostering positive communication Flexible and adapted to individual progress and parental learning styles	
Steps of PACT	
Follows a six-step approach aligned with child development to enhance fundamental socio-communicative skills Initial steps enhance parent–child connection, synchronization, and responsiveness. Subsequent stages develop the child’s expression and comprehension through appropriate language modeling. Progression through stages is based on predefined criteria emphasizing communication initiation, anticipation, routine, and language expansion for verbal children in later stages. Individual sessions occur biweekly for six months, then monthly for the next six.	
PACT in-center setting	Videoconferencing PACT (Aldred, 2022)
Duration: Each PACT teleconference session is approximately 60–90 min in length. Sequence: Beginning (10–15 min): review of progress and agenda setting (daily practice, recall of goals, exploration of changes) Middle (30–60 min in total): A 10-min parent–child play sessions is recorded and then watched with the therapist. The parent and/or therapist isolate short video clips to review in order to identify moments of interaction and clarify the parent’s positive role in this interaction. End (10–15 min): summary and home program discussion Homework: 30 min per days with PACT objectives in mind	Duration: Each PACT teleconference session is approximately 60 min in length. The 10-min parent–child play video is sent to the therapist before the session and parents are asked to watch it just before the session Sequence: Beginning (5–10 min): review of progress and agenda setting (daily practice, recall of goals, exploration of changes) Middle (20–30 min): The parent and/or therapist isolate short video clips to review before the session, aiming to identify moments of interaction and clarify the parent’s positive role in these interactions. End (10 min): summary and home program discussion Homework: 30 min per days with PACT objectives in mind
Evidence-based data	
In a British randomized controlled study (*N* = 152) including children aged 2 to 10 and comparing PACT intervention to standard care, PACT has shown a reduction in the severity of autism symptoms measured by the Autism Diagnostic Observation Schedule version 2 (effect size (ES) = 0.64; 95% CI 0.07–1.20) and an increase in parental communication synchronization with the child (ES = 1.22, 0.85 to 1.59) and child-initiated communication with the parent (ES = 0.41, 0.08 to 0.74) (Green et al., 2010; Pickles et al., 2016). The follow-up study demonstrated a lasting effect on autism symptom severity six years after the intervention, with an overall significant reduction in symptom severity during the trial and follow-up period (ES = 0.55, 95% CI 0.14 to 0.91, *p* = 0.004) (Pickles et al., 2016). “Mechanistic” studies have also confirmed that the distal effect of PACT therapy on autism severity measured by ADOS was mediated by the improvement in child-initiated communication, itself mediated by the improvement in parent–child synchronization, reinforcing the initial theoretical hypotheses of the PACT mechanism (Aldred et al., 2012; Pickles et al., 2015). No study has assessed the feasibility of remote-PACT so far.	

### 2.3 Measures and procedures

#### 2.3.1 Feasibility

The feasibility of the videoconferencing intervention was rated by a clinician (MB) on videos of the recorded remote therapy sessions between parents and professionals. Six videos were randomly selected from the 12 follow-up sessions. An assessment tool was designed for this study, based on the literature on telehealth (Taylor et al., 2015) and with the help of one PACT senior therapist. Five items were evaluated (see Appendix): audio quality, video quality, Internet network stability, the ability to share and watch the parent–child video simultaneously, and total session duration. Each item could score 1–4 points. A session was considered “feasible” if the score on the scale was equal to or greater than 15.

#### 2.3.2 Autism severity and parent–child interaction assessment

Every parent–child interaction video sent by the parents to the therapist was saved under a code name with a random number from 1 to 12. Every therapy session was registered and saved on a secured cloud storage system.

To evaluate autism symptoms, the Brief Observation of Social Communication Change (BOSCC) (Grzadzinski et al., 2016) was used and rated by a trained clinician (AJ), and the OSU-CGI-S (OSU Research Unit on Pediatric Psychopharmacology, 2005) was rated by two trained clinicians (MB and GB) blinded to the therapy progression. Clinical Global Impression-Improvement (OSU-CGI-I) (OSU Research Unit on Pediatric Psychopharmacology, 2005) was rated by a trained clinician (LJ) independent of the two other clinicians. The video was rated in order of dates for the rating of the CGI-I. The clinician rating CGI-I was not blinded to therapy progression.

To evaluate changes in parent–child interaction, the Dyadic Communication Measure for Autism (DCMA) (Aldred et al., 2012) was rated by a trained research assistant (SD).

### 2.4 Statistical analysis

Statistical analyses were conducted using JASP software (JASP Team, 2023). For continuous outcomes, means with standard deviations were calculated. The percentages were calculated for dichotomous outcomes. As this is an exploratory study, no sample size was calculated prior to the study, and the results presented here are only descriptive.

Eighty percent of the recorded debriefing sessions should obtain a score of 15 or more for videoconferencing intervention to be considered feasible.

The study was approved by the University of Lyon Institutional Review Board (IRB 2021-01-12-01) and the parents received written information.

### 2.5 Qualitative study

A qualitative study was conducted to understand the experiences of PACT therapists in delivering PACT through videoconferencing and identify both barriers and facilitators.

Data were collected through semi-structured interviews with the eight PACT therapists, by a master student, trained in the interview method. The interviews lasted 30–60 min and were audio-recorded, transcribed verbatim, and anonymized. The interview guide was developed by the authors following a literature review (Jurek et al., 2022; see Appendix).

A semantic thematic approach was used to analyze the data (Guest et al., 2012). All original recordings and transcriptions were analyzed in French. First, interview transcripts were read multiple times to allow researchers to familiarize themselves with the data. The transcripts were then inductively hand-coded, which allowed for the organization of recurring ideas into broader categories. Two researchers (MMB and LJ) independently coded the interviews using NVivo software (version 12) (QSR International Pty Ltd, 2020). Each interview transcript was repeatedly analyzed until no additional categories could be identified by the researcher. The categories created through this process served as the basis for the study’s themes and sub-themes. Discussions were conducted whenever there were disagreements in the coding process until a consensus was reached. To ensure investigator triangulation, a third research expert (MMG) was invited to review and refine the extracted codes, categories, and subcategories.

The themes and subthemes were then interpreted to reflect the key ideas underlying the participants’ responses in their interviews. The coders selected representative quotations to present the categories or sub-categories. The main results were sent to the interviewed therapists for validation, allowing them to confirm that the findings accurately reflected their experiences and perspectives.

The French results were translated into English and back-translated into French to ensure consistency.

#### 2.5.1 Reflexivity

The research team for the qualitative analysis consisted of three child and adolescent psychiatrists, two of whom specialize in autism. Two researchers were trained in the qualitative research. The third researcher clarified the results using the scientific literature.

## 3 Results

### 3.1 Feasibility

**Figure 1:**
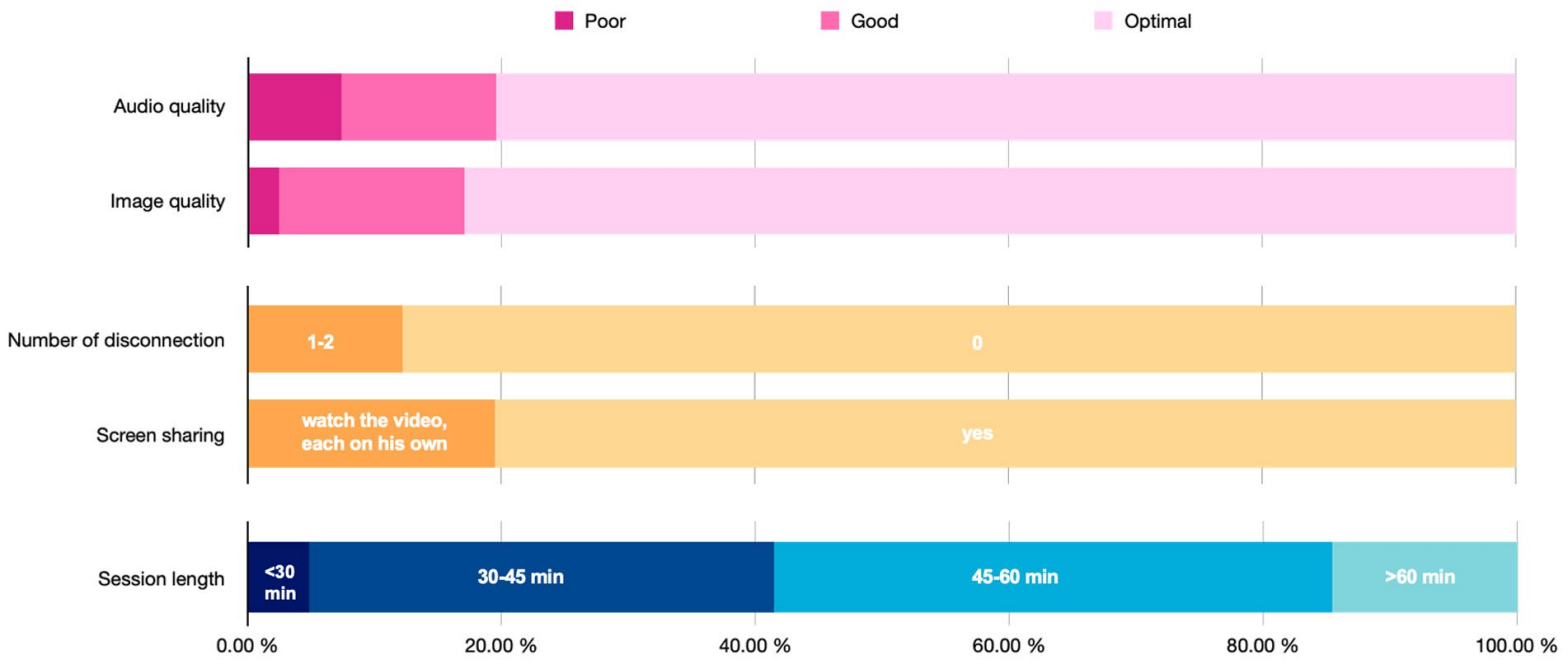
Feasibility of videoconferencing intervention.

From an expected total of 54 intervention videos, 41 videos were retrieved and rated. Thirteen sessions were not recorded and, therefore, missing. A score greater than 15 was obtained for 95.1% of the videos (Figure 1).

Difficulties were observed concerning the audio quality in 7.3% of the videos with an audio quality rated as “poor” and difficulties for the parent and the therapist to hear each other, or delay in the responses. In 19.1% of the sessions, screen sharing of the parent–child video was not possible, and the parents and therapists had to look at the video separately at the same time. Only 2.0% had poor video quality. The PACT sessions were shorter than in center setting with 37% of sessions lasting between 30 and 45 min, and 5% of the sessions less than 30 min.

### 3.2 Changes during intervention

Changes in autism severity and child–parent interaction are presented in Table 2.

**Table 2:** Changes in autism severity and in parent interaction during intervention for the nine children included.

Scales (min-max)	Mean at baseline (SD)	Mean at mid-therapy (SD)	Mean at endpoint (SD)
BOSCC (0–60)	25.5 (10.4)	27.3 (12.5)	20.5 (13.6)
DCMA parent–child synchrony*	27.6 (10.7)	32.9 (18.8)	33.1 (10.5)
DCMA child communicative initiation*	7.5 (12.0)	15.8 (12.6)	16.9 (17.2)
DCMA mutual shared attention (0–100)	76.0% (12.7)	77.3% (19.1)	86.6% (15.1)
CGI-S (1–7)	4.7 (1.3)	4.7 (1.4)	4.2 (1.4)

SD, Standard Deviation; BOSCC, Brief Observation of Social Communication Change; DCMA, Dyadic Communication Measure in Autism; *number of acts.

Concerning the measure of global change with the CGI-I, one child was considered very much improved, two were considered much improved, three were considered minimally improved, and two had no change in global symptomatology.

### 3.3 Barriers and facilitators of videoconferencing intervention: professionals’ point of view

Our thematic analysis yielded four main themes. Themes and subthemes are presented in Table 3. Detailed results and quotes are presented in Supplementary Table S1.

**Table 3:** Summary of findings: therapists’ experience with video-PACT.

Summary of findings: themes and subthemes
Positive changes on many levels Increases parents’ confidence in their abilities Change in parent’s position in interaction with the child Allows parents to take time specifically for their child PACT enables parents to adjust their expectations to the child’s rhythm. The therapist’s position as a facilitator of change is a satisfying one. PACT enables the therapist to learn to better adapt to the parent’s profile. PACT enables the therapist to develop a clinical viewpoint more focused on the interaction and its development. Impact on the child, beyond play Significant and persistent results for a small number of sessions
Intervention may be affected by certain factors A higher flexibility in the different stages of PACT could allow for better accommodation to parents’ and children’s needs Complementary techniques may be required Impact of the parent’s profile on treatment Homework time as an obstacle to intervention
Videoconferencing, a tool that can be implemented under certain conditions More ecological intervention with video The benefits of videoconferencing for intervention implementation Therapeutic relationship possible in visio Intervention by videoconference is dependent on technical aspects but remains feasible PACT through videoconferencing allows the therapist to anticipate and prepare sessions well
The essential components of PACT Requires a great deal of thought and analysis on the professional’s part Importance of PACT structure and intervention schedule Importance of video feedback Implementing the objectives in day-to-day life rather than in the daily 30-min homework Requires significant parental investment Importance of preparing parents for PACT intervention

See Supplementary Table S1 for detailed results.

#### 3.3.1 Positive changes on many levels

Therapists explained that PACT has a multifaceted impact on parents, children, and therapists alike. PACT boosts parental confidence, particularly in their parenting skills. Therapists observed a shift in parents’ position during interactions with their children, characterized by increased synchrony and a deeper connection. They described PACT as a means for parents to dedicate a specific time to their children. PACT appeared to enhance parents’ ability to adjust their expectations to their child’s unique rhythm.

For therapists, PACT serves as a transformative tool that positions them as facilitators of change in a fulfilling manner. Through this therapy, therapists gain insights into better tailoring their approach to match each parent’s individual profile, cultivating a clinical perspective centered on interaction and its evolution.

Remarkably, the impact on child behavior extends beyond mere play, yielding significant and lasting results within a relatively small number of sessions.

#### 3.3.2 Intervention may be affected by certain factors

Therapists claim that the success of interventions depends on multiple factors. An increase in flexibility throughout PACT’s stages would significantly improve the program’s ability to cater to the unique requirements of parents and children. Some stages may not align perfectly with the child’s needs and could potentially lead to a decrease in parental motivation.

Similarly, complementary techniques may also be necessary to address unique challenges. Some parents may require assistance with aspects that are not directly addressed by PACT, which can occasionally interfere with the effectiveness of PACT therapy, thus necessitating support from other sources.

The impact of a parent’s individual profile on the treatment process should not be underestimated as it requires further adaptation from the therapist. Furthermore, the allocation of homework time may sometimes act as an obstacle to the progress of the intervention.

#### 3.3.3 Videoconferencing, a tool that can be implemented under certain conditions

Videoconferencing, while a valuable tool, can be successfully implemented under specific conditions according to therapists’ experience. It presents a more ecological approach that allows parents to participate in a familiar environment, where they can feel more secure but also reduces the need for physical travel and can better align with parents’ schedules. Therapists have confirmed the possibility of therapeutic relationships using videoconferencing.

However, the success of interventions through videoconferencing is contingent upon addressing technical aspects, ensuring a feasible connection. On the professional side, implementing PACT through videoconferencing demands greater organization but allows the therapist to anticipate and prepare the sessions well.

#### 3.3.4 The essential components of PACT

PACT was described as a useful therapy when the essential components are respected. Implementing a PACT intervention successfully requires a great deal of thought and analysis of the professional’s part to maintain fidelity to the PACT model. Therapists have highlighted the importance of the PACT structure and schedule, which serves as a framework for consistent progress and seems to be acceptable for both parents and professionals. The use of video feedback has been described as a crucial element in PACT, facilitating parents to progress at their own pace and empowering them to independently identify acceptable solutions to aid in their child’s development. Therapists encourage parents to apply the intervention’s objectives in their day-to-day lives rather than limit them to the prescribed 30-min homework sessions, aiming to enhance acceptability while maintaining consistency in the work. PACT Therapists underlined the importance of adequately preparing parents for their participation in the PACT intervention as it requires significant parental investment.

## 4 Discussion

Our findings suggest that PACT intervention conducted through video conferencing is feasible even in the face of technical issues reported during feasibility assessments and interviews. The therapist reported some difficulties due to poor audio quality on some computers but also in watching the video together with the parent by sharing their screen. Most technical difficulties appeared to be related to the instability of the network used by the participants, particularly those living in rural areas. Nevertheless, the qualitative study indicated that therapists found different ways to overcome technical difficulties and to preserve the quality and main components of the intervention (e.g., watching the video simultaneously on separate screens, using a parallel phone call to ensure better audio quality). The sessions were shorter than those in the center setting. This result is mainly explained by the organization of PACT in videoconferencing, where the parent–child video was recorded and watched by a professional once before the videoconferencing session (Aldred, 2022).

The main component of PACT was adequately transposed to the remote PACT. The establishment of a therapeutic relationship via video conferencing was successful; therapists and parents worked to improve parent–child synchrony, a main moderator of PACT efficacy, using video feedback. This highlights the adaptability of therapeutic interactions to remote platforms, thereby expanding the accessibility of interventions, such as PACT. The remote format also provided ecological benefits, such as tailoring interventions to parents’ needs and minimizing logistical challenges.

Child development and parent–child interactions showed positive changes in various aspects, as supported by the qualitative analysis results. These findings suggest some potential benefits of PACT, including enhancing parents’ confidence in their parenting. The findings align with previous qualitative studies on parent-mediated interventions (Jurek et al., 2022; Leadbitter et al., 2020; Pickard et al., 2016).

However, this study has some limitations. The absence of a control group and the small local sample size are notable constraints that reduce the generalizability of the findings. We were unable to retrieve certain session videos to assess feasibility, which may be related to technical issues during the sessions.

Concerning the qualitative study, the use of a convenience sample is a limitation as we had a limited number of participants. Nevertheless, no new themes were found after the 6^th^ interview, and our population had sufficient variation to cover multiple experiences with PACT.

We acknowledge that not exploring parents’ perceptions of the PACT therapy delivered via videoconference represents a limitation of our study. However, our ongoing randomized controlled trial includes a qualitative component that will specifically address parents’ perspectives on this aspect of the intervention.

## 5 Conclusion

This preliminary study suggests that PACT intervention using videoconferencing is feasible despite technical concerns. The observed enhancements in parent–child interactions and the positive outcomes developed in the semi-structured interviews offer encouraging implications for remote interventions. Further research is required to confirm these results. A multicenter randomized trial is planned for this matter (Jurek et al., 2021).

## Data Availability

The datasets presented in this article are not readily available because the authors do not have the right to share the data. Requests to access the datasets should be directed to Lucie Jurek, lucie.jurek@ch-le-vinatier.fr.
