# Supplementary material for: Feasibility of a videoconferencing-based parent-mediated intervention: a mixed-method pilot study
Source: Front Psychol. 2025 Jan 7;15:1450455. doi: 10.3389/fpsyg.2024.1450455 (PMC11753217; doi:10.3389/fpsyg.2024.1450455)
Supplement: Supplementary file 1 [file Table_1.DOCX]

**Supplementary Table 1.** **Results of the qualitative study: Therapists’ experience with video-PACT.**

| **Themes and subthemes** | **Meaning** | **Quotes** |
| --- | --- | --- |
| **Positive changes on many levels** | | |
| Increases parents' confidence in their abilities | The parents conveyed the realization that they had numerous skills to support their child. They mentioned to the therapists the sense of regaining parental competence that was lost during the diagnostic wandering phase. | « It's a valuable support, because it serves beyond the communication goals, which are extremely important, but it also serves parents to increase a sense of effectiveness. » |
| Change in parent's position in interaction with the child | The therapists were able to observe parents who were more attuned to the needs and desires of their child. There was a willingness to rediscover the joy of shared experiences with the child, while actively responding to their needs. | « I have seen significant behavioral changes in the way parents interact with their children, i.e. being able to adjust more to their child. » |
| Allows parents to take time specifically for their child | The PACT therapy enables parents, caught up in busy daily routines, sometimes with other children, to take a few moments to focus on the child's needs, both through homework and during sessions with the therapist. | « Understanding autism for their child on a truly individual level, taking time for their child and truly being there for them. » |
| PACT enables parents to adjust their expectations to the child's rhythm. | The parents demonstrated an adjustment of their expectations to the reality of their child. These more realistic expectations enable parents to anticipate their goals, leading to the rediscovery of positive and encouraging interactions. This adjustment contributes to a sense of calmness in the parent-child relationship. | « It has also enabled them to understand […] how their child interacts specifically and to adapt to their child's pace. This is extremely important. And revising their expectations and the pace they set for him, so they can really adapt.» |
| The therapist's position as a facilitator of change is a satisfying one. | The PACT therapist serves as a facilitator of change, adopting a less paternalistic stance that allows the therapist to feel a sense of effectiveness. | « For me, it has really taught me to question people in a different way, not to tell people what I think is good, but for them to say it and for it to come from them. And I find that empowering as a professional. » |
| PACT enables the therapist to learn to better adapt to the parent's profile. | Training in PACT allows professionals to take the time to adapt to the specific needs of individuals. While it has specific objectives, the step-by-step nature of this therapy serves as a learning experience for professionals, influencing their practice beyond PACT. | « In terms of psychoeducation, it allows for better adaptation to the parents' profile: their communication profile, their interaction profile, and being more attentive. » |
| PACT enables the therapist to develop a clinical viewpoint more focused on the interaction and its development. | Training in PACT allows professionals to approach the socio-communicative development of the child in a more positive manner, being sensitive to all communication signals. It enables professionals to view autism in terms of possibilities for the child, rather than merely as symptoms to be treated. | « From a professional perspective, it has taught me a lot in terms of clinical skills, interview management, and sensitivity in observing communication. » |
| Impact on the child, beyond play | Professionals were able to describe numerous impacts on the socio-communicative skills of children, i.e. the quality of social interactions, the quantity of initiation, the adaptative competencies. | « It unlocked many things [initiation] in these individuals, even though initially, we were just playing games, it had a ripple effect on their whole lives. » |
| Significant and persistent results for a small number of sessions | Even though PACT is delivered in a less intensive manner to parents, the effects observed by professionals are significant and enduring, especially during booster sessions. | « And for the family, I think that for the number of sessions we do, it's very powerful. […] there are a lot of results for twelve sessions. » |
| **Intervention may be affected by certain factors** | | |
| A higher flexibility in the different stages of PACT could allow for better accommodation to parents’ and children’s needs | The intervention, intended to be flexible, could be even more relevant for families if the steps could be adapted, as some parents found the adherence to specific steps to be a barrier. This was particularly true when the chosen objectives did not always seem to correspond to the needs of the child. | «In the current method, you are supposed to follow step 1, step 2, step 3, etc., in order. I think we could offer a little more flexibility [based on] what we believe needs to be worked on first. » |
| Complementary techniques may be required | The PACT is not an intervention that addresses all the challenges inherent in autism. It should be integrated into a comprehensive care program for the child, including individual and group interventions targeting behavioral issues and psychoeducation. When the PACT is the sole intervention, parents may be disappointed as it does not meet all their expectations. The presence of behavioral issues can be a barrier to the success of the therapy. | «For me, as a therapist, PACT cannot address everything. It primarily focuses on the social interactions between the parent and child dyad. So, first of all, a PACT approach is interesting, but it will have to be followed up by other types of intervention. » |
| Impact of the parent's profile on treatment | The PACT is a reflective therapy that taps into the resources within the parent. Some parents find it challenging to embrace this reflective approach and instead expect direct guidance from the therapist. Additionally, some parents anticipate a more behavior-focused therapy and may feel disoriented by the developmental aspect of the PACT. | «And then, there are people who struggle to answer questions. Some find it difficult to talk extensively to express their thoughts. For some, especially those in denial, it can be challenging. » |
| Homework time as an obstacle to intervention | One of the most challenging aspects for therapists is to ensure that parents complete the homework assignments. Parents have mentioned the difficulty of remembering these tasks during the day, especially with busy schedules or fatigue after a day filled with various activities. It is challenging for professionals to emphasize this point without making parents feel guilty. | « I find that families still seem to have difficulty integrating it into their daily lives, based on what they tell us. It's more like, 'Oh yes, I played with him, but I didn't necessarily think about it.'» |
| **Videoconferencing, a tool that can be implemented under certain conditions** | | |
| More ecological intervention with video | The intervention is perceived as more natural by parents through video sessions. They are in their chosen environment, feeling more comfortable. They can also choose toys with which they are familiar and will engage with again. The intervention becomes more aligned with their daily life. | « While here, we are in their living environment, with their parents, and with the games that the parents have selected. So, it's incredibly interesting because it really provides a better understanding. It almost gave me the impression of being more effective. » |
| The benefits of videoconferencing for intervention implementation | Videoconference intervention helps overcome implementation barriers. Parents can choose the right time in relation to work, other children, without worrying about travel. | « In fact, my main point is that it makes people's lives easier. Because they don't have to travel, for people who work, you can squeeze them in between. It only takes an hour, in the end; whereas otherwise, they've got the journey, they've got everything.... » |
| Therapeutic relationship possible in visio | The therapeutic relationship remained unchanged during videoconferencing, with connections that could be established as early as the first session. Technical issues with videoconferencing may have made some sessions challenging but did not have a significant impact on the overall therapy. Professionals noted that when the parent was in their own environment, the relationship became almost more intimate. | « If the connection works well, these relationships start directly through video. In fact, we manage to create a good therapeutic alliance despite everything. » |
| Intervention by videoconference is dependent on technical aspects but remains feasible | Technical issues were reported with video, the most challenging being the inability to share and watch the video together. However, therapists quickly found technical solutions, sometimes with the assistance of parents. Parents demonstrated strong adaptability during technical challenges. Video conferencing has become a common aspect for parents. | « Yes, bugs can happen. I've had a few, especially with screen sharing [...] But in the end we always manage to get by. For example, the mom watched the video the minute I told her to. » |
| PACT through videoconferencing allows the therapist to anticipate and prepare sessions well | Having the parent-child video before the session allowed professionals to prepare for the session and select crucial moments for the parent. While this may lead to a loss of spontaneity, such preparation helps target the most positive moments for the parent to focus on their skills and make positive aspects evident. | « And I think it's a good idea to have the time to see the video sent by the parent beforehand, to prepare the session properly, than when it's live, when you film the child straight away and then go over the film with the parent. » |
| **The essential components of PACT** | | |
| Requires a great deal of thought and analysis on the professional's part | Since the PACT changes the therapist's approach, who is typically trained in more directive care, the therapy requires therapists to continue their education, undergo supervision, and share their concerns with other trained colleagues. This ensures that therapists can effectively support parents without taking over their roles. | « So it's care that's... I was going to say "tiring", but it requires professionals to put a lot, a lot of thought and analysis into getting parents to reflect. » |
| Importance of PACT structure and intervention schedule | The routines of PACT therapy, driven by its structure, provide parents with essential reference points to understand the objectives and stay motivated. This structure also allows parents to remain creative and natural during interaction times, which is not always achievable in more learning-focused therapies. | « For parents, it's somewhat the same. That is, it's also structured, they know where they're going, we always do a bit of the same thing, and in this routine, they have the freedom to think about their relationship with their child. » |
| Importance of video feedback | The video feedback was widely reported as a crucial aspect of the therapy. Therapists could recount beautiful moments shared with parents, discovering on the screen their abilities to assist their child. They explained it was crucial to be able to watch the video together. | « There's the impact of the video, which is inherently very objective, so they see themselves in action, and it's very powerful, the video... I was already using it before, but now, showing them the positive moments, it's... yes, it's really strong for the parents, I think. » |
| Implementing the objectives in day-to-day life rather than in the daily 30-minutes homework | It is essential to guide parents in completing assignments at home. Some parents are not accustomed to taking the time to play with their child, and it is the therapist's responsibility to work with the parent to find ways to implement the advice discussed in sessions into daily routines. This ensures that the child benefits from the intervention consistently. | « It's important to emphasize what these thirty minutes should be, in fact. To really provide ideas on how they can integrate it into their daily live. » |
| Requires significant parental investment | Therapists have observed that families achieving the best results with the intervention are those who invest the most. The therapy does not benefit parents as much if they do not complete the homework and only focus on the techniques highlighted during the 10-minute video sent to the therapist. | « The family has to invest in it, and people have to want to come, otherwise it doesn't work. » |
| Importance of preparing parents for PACT intervention | The PACT is particularly effective and works well with parents who are well-prepared for the intervention. Ideally, those who have already received psychoeducation between the diagnosis and the intervention, and for whom the specific goals of the PACT have been explained and understood. Inappropriate expectations for therapy make parents' motivation much more variable. | « It has to be care that's well, well explained to parents beforehand, because often what parents want is for us to take care of their child, not them. The introduction of this treatment should also be done in a very specific and well-structured framework, because otherwise, you can quickly find yourself in the first session with parents who haven't understood that we will never intervene directly on their child. » |
