# Supplementary material for: Feasibility of a videoconferencing-based parent-mediated intervention: a mixed-method pilot study
Source: Front Psychol. 2025 Jan 7;15:1450455. doi: 10.3389/fpsyg.2024.1450455 (PMC11753217; doi:10.3389/fpsyg.2024.1450455)
Supplement: Supplementary file 2 [file Table_2.DOCX]

**Appendix**

**Technical feasibility assessment tool**

| Item | 4 | 3 | 2 | 1 |
| --- | --- | --- | --- | --- |
| Audio quality | Optimal | Rather good | Mediocre | No audio (use of another way to communicate) |
| Video quality | Optimal | Rather good | Mediocre | By audio only |
| Number of disconnection | 0 | 1-2 | 2-3 | 4 or more |
| Sharing screen | Yes, shared on screen | No, watch the video separately at the same time | Video seen before the session | No video available |
| Duration | ≥60 min | 60-45 | 45-30 | ≤30 min |

**Semi-structured interview guide**

1. Could you tell me about your first PACT videoconference session?
   1. Was it different from the other PACT sessions you had conducted before? How?
   2. Do you think the intervention and its results would have been different if you had met the parents in person?
   3. Based on your experience, do you have any advice on using videoconferencing in parent intervention?
2. What are your overall thoughts on PACT therapy? And on the time the intervention requires?
3. In your opinion, what are the positive aspects of this intervention? For you as a therapist? For the parents? Do you have a positive memory related to PACT during the IFPAD study to share?
4. In your opinion, what are the negative aspects or challenges of this intervention? For you as a therapist? For the parents? Can you think of any obstacles to the PACT intervention? What has prevented you from progressing with a family in PACT?
5. According to you, what changes does PACT bring about for the families participating?
6. Do you plan to change your approach to practicing PACT in the future? How? Will you continue?
